# Repeat Lumbar Puncture: CSF Lactic Acid Levels are Predictive of Cure with Acute Bacterial Meningitis

**DOI:** 10.3390/jcm2040328

**Published:** 2013-12-17

**Authors:** Burke A. Cunha

**Affiliations:** 1 Infectious Disease Division, Winthrop-University Hospital, Mineola, NY 11501, USA; E-Mail: bacunha@winthrop.org; Tel.: +516-663-2505; Fax: +516-663-2753; 2 School of Medicine, State University of New York, Stony Brook New York, NY 11501, USA

A common clinical problem concerns the utility of repeat lumbar puncture (LP) in adults with acute bacterial meningitis (ABM), e.g., pneumococcal meningitis [1]. An LP is initially done for diagnostic purposes in patients with suspected ABM, *i.e.*, diagnostic lumbar puncture (DLP). A repeat LP (RLP) may be done 1–3 days after the initial DLP, if the patient shows no improvement. If a patient with ABM is not doing well after three days, adequacy of antimicrobial therapy is the main concern. Other reasons for RLP is to detect possible intracranial complications of ABM unrelated to adequacy of therapy [1,2]. 

Cerebrospinal fluid (CSF) Gram stain and culture aside, the single most important CSF diagnostic parameter of ABM is the CSF lactic acid level [3,4,5,6], even with a negative CSF Gram stain. While the usual CSF lactic acid level breakpoint is 2.2 mmol/L, for ABM a cutoff of 6 mmol/L is highly sensitive/specific and rapidly/accurately differentiates ABM from partially treated bacterial meningitis (PTBM), as well as aseptic acute meningitis/encephalitis [6]. Typically, initial DLP CSF lactic acid levels in *S. pneumoniae* ABM are highly elevated (>12 mmol/L). Equally important, should the CSF lactic acid levels obtained on RLP have greatly decreased, this is predictive of cure. In the case of ABM, the most important CSF as predictor of therapeutic efficacy and good prognosis are CSF glucose and CSF lactic acid levels [6]. The CSF white blood cell (WBC) count and differential counts are much less sensitive predictors of therapeutic efficacy, as CSF pleocytosis may persist for weeks despite effective antibiotic therapy.

Antibiotic penetration into CSF depends on the antibiotic’s molecular size, lipid solubility and degree of blood brain barrier (BBB) permeability. The best index of BBB penetrability is CSF albumin, *i.e.*, the higher the CSF albumin, the greater BBB penetrability [7]. This is clinically relevant since some pneumococcal strains may be penicillin resistant *S. pneumoniae* (PRSP), and are often empirically treated with “high dose” vancomycin. In spite of the “high dose” vancomycin, CSF penetration is not assured as only ~15% of vancomycin simultaneous serum levels penetrate the CSF in the presence of inflammation, *i.e.*, ABM and <1% penetrates the BBB in the absence of inflammation, *i.e.*, no ABM. Therefore, increasing the dose of an inflammation dependent antibiotic, e.g., vancomycin, is likely to be ineffective in the case of PRSP ABM. In contrast, “meningeal doses” of third generation cephalosporins or meropenem penetrate the CSF if PRSP is administered in therapeutic concentrations [8].

In ABM, RLP with a marked decrease in CSF lactic acid levels, is predictive of cure. Clinically this is important since the CSF Gram stains may remain positive for RLP [1]. A persistently positive Gram stain for RLP may mislead any physician unaware of the problem into changing therapy, but markedly decreased CSF lactic acid levels are predicative of the therapeutic effectiveness of the cure and antibiotic [6,7,9].

Unless the patient is clearly not improving or has even deteriorated, RLP is usually not necessary. CSF lactic acid levels are helpful both diagnostically in the DLP and prognostically in the RLP, and helpful in confirming whether antimicrobial therapy is effective and predictive of cure. The treatment for “penicillin resistant” pneumococci (PRSP) remains as penicillin (or a β lactam given in “meningeal doses”). In contrast to vancomycin, in “meningeal doses” of third generation cephalosporins, e.g., ceftriaxone 2 g (IV) q12 h, the penicillin non-susceptibility and resistance breakpoints for PRSP are well below achievable CSF concentrations [10]. CSF levels following 2 g of ceftriaxone are approximately 257 mcg/mL which is well above the minimal inhibitory concentration (MIC) of even highly resistant (PRSP) in CSF [8].
